# Iconic buildings in the making of city identity: The role of aspirational identity artefacts

**DOI:** 10.1177/00420980221144157

**Published:** 2023-01-26

**Authors:** Alessandra Zamparini, Gastone Gualtieri, Francesco Lurati

**Affiliations:** Università della Svizzera italiana (USI), Switzerland; Università della Svizzera italiana (USI), Switzerland; Università della Svizzera italiana (USI), Switzerland

**Keywords:** city identity, iconic buildings, qualitative, urban planning

## Abstract

Iconic buildings are important meaning generators in cities. This study explores the role that iconic buildings *in-the-making* have in the discursive construction of city identity in public debate. Through the examination of the Locarno PalaCinema case (Switzerland), our study proposes that iconic buildings – during their planning – can serve as aspirational identity artefacts: objects that are mobilised in discourse to inform productive idealisations of city identity by powerful urban actors. Findings identify the mechanisms through which the aspirational artefact and city identity interact in discourse, showing that iconic building projects *orient* city identity claims, while at the same time city identity meanings taken from collective memory, present understandings and future aspirations are used by actors to *infuse* the evolving project with meaning. This study aims to contribute to debates in urban planning and city identity by discussing the identity anticipation role of the planning of iconic buildings and how they can be a productive ground to reflect, re-orient and re-claim the unique features of a city’s identity while aspiring to achieve a different future.

## 摘要

标志性建筑是城市中重要的意义生成器。本研究探讨了在公众的辩论中，正在建设中的标志性建筑在城市身份的话语建构中所起的作用。通过对 Locarno PalaCinema 案例（瑞士）的考查，我们提出，标志性建筑在其规划过程中可以作为理想的身份制品：强大的城市行为者在话语中调动这些对象，解释富有创造性的、理想化的城市身份。我们的研究结果确定了理想的制品和城市身份在话语中相互作用的机制，表明标志性建筑项目对城市身份主张进行定向，同时，行为者利用从集体记忆中获得的城市身份的意义、当前的理解和未来的愿景赋予不断发展的项目以意义。本研究旨在通过讨论标志性建筑规划在身份预期方面所起的作用，以及它们如何成为反映、重新定位和重新获得城市形象独特特征的富有成效的基础，为城市规划和城市形象的讨论做出贡献，同时致力于实现不同的未来。

## Introduction

Iconic buildings – namely, new major architectural projects that provide the city with regenerating power because of the attention they receive (Jencks, 2006; Raevskikh, 2018) – play an important role in the social construction of modern urbanity (Gospodini, 2004; Sadana, 2018). They contribute to shaping new labels and images of the ‘entrepreneurial city’ (Nagel and Satoh, 2019: 1682) ‘creative city’ (Scott, 2014: 569), and sustainable city (Kornberger et al., 2021). Iconic buildings, together with other elements of a city’s built environment, are also relevant in representing the city symbolically and shaping perceptions about the distinctiveness of cities. Their aesthetics and materiality can serve as powerful collective meaning generators, informing perceptions of visitors and locals about the unique elements of a city’s character (Jones and Svejenova, 2018; Kavaratzis and Hatch, 2013), but also contribute to the emergence of new and divergent interpretations (Gospodini, 2004).

Although most research focuses on iconic buildings’ effects after their construction, some scholars suggest the potential for exploring their ‘anticipation effects’, referring to the demographic and economic dynamics they spark during their creation (Raevskikh, 2018). We know less about the socio-cultural dynamics, however, that activate during the often long and contested path of ideation, approval and realisation of such works (Petani and Mengis, 2016; Sadana, 2018). The case of the construction of the Locarno PalaCinema provides a particularly revelatory context that invited us to delve into those socio-cultural dynamics by investigating a different type of anticipation effect, concerning the discursive (re)construction of a collective sense of city identity.

Locarno is a small Swiss city on the shores of Lake Maggiore known for its enchanting landscape and sunny weather. The city and its surrounding region are also known for being the home of the Utopian cultural movements in the late-19th and early-20th centuries that created an environment attracting artists and intellectuals. The aura of Locarno was instrumental in the decision to hold peace negotiations there in 1925, known as the Locarno Treaties, when Harold Nicholson, a British diplomat, talked of ‘the Heavenly alchemy of the Locarno spirit’ (Gibbon and Finn, 2015). This spirit contributed to the inspiration that led to the creation of the Locarno Film Festival two decades later, which today is among the major international film festivals and home of the auteur cinema community. Despite all that, at the beginning of the 2000s, Locarno’s identity traits, as a town of peace, international gathering and of vanguard artists and intellectuals, were rarely mentioned in the public debate. Relegated to the history books, they were seldom remembered as elements of pride (Volonterio, 1997). In addition to its tourist identity, Locarno had a strong but controversial link with the Locarno Film Festival. For some, the festival is ‘an element of disturbance for a quiet reality that has other things to think about’; for others, the festival is a dream that ends when Locarno goes back to the quiet provincial life of a small Swiss town (Ambrosioni, 1998: 36). The relationship with the festival effectively represents the ambivalent relationship that Locarno has had with its cultural side and the ‘cultural myopia’ that, according to many intellectuals, has characterised the local government for the second half of the 20th century (Volonterio, 1997: 71). Something changed dramatically with the decision to build a permanent house for the festival – an iconic architectural project later renamed PalaCinema – worth 33 million Swiss francs. During the development of the project, culture, cinema and audio-visual education became increasingly relevant distinctive features describing Locarno’s identity in public debate.

Inspired by the empirical phenomenon we were observing, we asked: How does engaging in the planning of an iconic building interact with a city’s discursive identity construction in public debate? We thus developed a longitudinal qualitative discourse analysis to examine how this happened and track the evolution of city identity discursive articulations by politicians, planners, journalists and local opinion leaders.

The paper is organised as follows. First, we review the theoretical definitions of city identity informing our study as well as in relationship with the built environment and urban planning. Then, after providing further details on our case and methods, we present the findings and discuss them in light of current debates in urban studies.

## Theory

### City identity as a discursive construction

Defining city identity is a challenging endeavour. The identity of a city – like other space-bounded collective identities – could be defined by its ‘“natural” or “essential” characteristics’ (Cerulo, 1997: 386), such as physical, historical and cultural attributes, yet a great part of the current sociological and geographical debate suggests that city identity can be best understood as the result of a social construction, an ‘imagined community’ (Anderson, 1991) or ‘social artifact’ (Cerulo, 1997: 390) emerging from the ways actors interpret, narrate, symbolise and manipulate its unique character or spirit (Jones and Svejenova, 2018; McCarthy, 2006; Paasi, 2013).

According to this view, a city’s identity, ‘by means of language and other semiotic systems, [is] produced, reproduced, transformed and destructed’ (De Cillia et al., 1999: 153). In other words, it is not simply revealed and neutrally represented, but actively brought into existence (Bourdieau, as cited by Paasi, 2003) by various actors’ discursive articulations. These discursive articulations contribute to the dynamic emergence of a collective sense of self by continuously providing (re)interpretations of a shared collective past, present and desired future; by delineating and shifting boundaries between we-ness and otherness in urban developments; and by highlighting certain meanings while eliding others (Anderson, 1991; De Cillia et al., 1999; Jones and Svejenova, 2018; McCarthy, 2006; Paasi, 2013). Therefore, multiple voices and perspectives contribute to the collective emergence of the distinctive character of a city. However, institutional voices such as politicians, intellectuals, the media and urban planners are widely recognised as having more power in characterising this collective sense of self through their discursive ‘moulding’ of a city’s identity in the form of narratives, rhetorical acts and explicit claims (Paasi, 2013: 1214; see also Jones and Svejenova, 2018; McCarthy, 2006). These characterisations are more visible and, although they may be resisted by other perspectives, such as citizens’ activists, they are in general more influential in shaping perceptions of both local and external actors (De Cillia et al., 1999; Paasi, 2013).

In this respect, it is useful here to specify that the conceptualisation of city identity provided herein corresponds to an understanding of the identity *of* a place, which is different from the socio-psychological notion of place identity at the individual level – meaning ‘the identification of people with a place’ (Cerulo, 1997; Paasi, 2013: 1209). Although we acknowledge that, in a dynamic understanding of the phenomenon, discursive claims do influence individual perceptions and the development of a sense of identification, in this study we specifically focus on the former.

Similarly, we would like to distinguish the notion of city identity from the one of city branding. Branding differs from identity insofar as it refers to purposeful strategic symbolic constructions geared towards positioning a place in the minds of external stakeholders in particular (Richards and Wilson, 2004). Although identity is certainly dynamically related to branding, providing the ‘raw material’ upon which city brands are developed (Kavaratzis and Hatch, 2013: 82) and being sometimes commodified (Paasi, 2003) in those strategic exercises, not all identity discursive articulations, even the most influential, are deliberately developed for marketing purposes.

Unlike strategic city branding, the discursive construction of identity is a long-term and rarely linear ‘accumulation’ of meanings (Paasi, 2013: 1208). Indeed, city identity may be taken for granted and remain relatively dormant in the public debate, then suddenly reactivate when specific contextual factors require, appealing to a shared sense of self (McCarthy, 2006). Indeed, this is what happened in the Locarno case with the decision to engage in the construction of an iconic building. In the next section we provide a brief illustration of the literature addressing the relationship among built environment, planning and identity.

### Built environment and discursive interpretations of the past, present and future city identity

The built environment, such as monuments, parks and architecture in general, includes relevant resources for the discursive construction of a city’s identity in multiple ways. First, historical buildings work as ‘reminders of the past’ for citizens (Main and Sandoval, 2015: 75); in certain cases they provide tangible cues for commemoration, triggering heated conversations about a city’s collective memory (Halbwachs, 1992; Krzyżanowska, 2016).

Second, buildings provide material cues for developing interpretations of present city identities. In this sense, the built environment does not neutrally carry meanings of the past, but – as Jones and Svejenova (2018) suggest – interacts with discursive practices that decode the meanings they represent in the present and, at times, inscribe them with new meanings. A city’s architecture is, in fact, often decoded through rhetorical acts that define a city’s identity by using a part to represent the whole (e.g. referring to Barcelona as ‘Gaudi’s city’ because of specific buildings) or by emphasising some traits and silencing others (e.g. Wall Street makes New York a financial capital and silences the definitions that more problematic areas may suggest).

Third, and much less explored, the built environment may materially provide cues to imagine possible (un)desired future identities. In particular, iconic buildings are monuments to the future (Jencks, 2006) representing visions of hypermodern society, such as the Guggenheim Museum in Bilbao (Gospodini, 2004), offering inspiration to citizens for ‘new and divergent interpretations’ of the city (Gospodini, 2004: 234–235) and individual ‘aspirations of upward social mobility’ (Sadana, 2018: 189), but also posing threats of hyper-conformity to modernity imperatives and loss of cities’ cultural identity (Anzoise et al., 2020).

Although several studies have explored the relationship between existing buildings and actors’ discursive articulations of city identity, we know less about how this relationship unfolds in the pre-construction phase (e.g. initial design proposals, architectural project approval) or actual construction. Spatial planning studies have suggested that planners engage with shared memories and future imaginaries (Comi and Whyte, 2018; Petani and Mengis, 2016; Quitzow and Rohde, 2021). Urban policymaking debates have suggested that the notion of identity is mobilised to activate different – and sometimes conflicting – discursive frames (Farhat, 2015; Paasi, 2013) while informing or opposing resistance to urban transformation (Leixnering and Höllerer, 2022). They discuss that identity, under different perspectives, is somehow connected to urban material policies and transformations, and, in turn, engaging in urban transformations seems to favour the emergence of a collective sense of we-ness (Kornberger et al., 2021). However, it remains unclear how past-, present- and future-oriented meanings that define a city’s unique identity are articulated and negotiated while planning relevant urban material transformations.

## Methods

This study aims to explore how engaging in the planning of an iconic building interacts with a city’s discursive identity construction in the public debate. It does so through the development of a longitudinal qualitative discourse analysis in the context of the Locarno PalaCinema case.

### Case study

Locarno (approximately 15,000 inhabitants or 50,000 if considering the larger urban aggregation) is the third largest city in the Italian part of Switzerland. Despite being known, both nationally and internationally, as home to the Locarno Film Festival (henceforth LFF), Locarno had no permanent infrastructure to accommodate it. The organisation of the 10-day festival required the necessary spaces to be activated each year and then dismantled at its end. Even LFF administrative offices, which employed about 15 permanent employees, were rented in an anonymous building. This lack became a pressing issue in the early 2000s and a topic of public and political debate because of two contingent facts. The LFF experienced dramatic expansion (Buccella, 2014), with the growing number of visitors and movies shown, and the infrastructural issue was ‘an objective limit’ (OB, 2005). This sustained growth took place during a decade of economic slowdown in the city characterised by the closure of several hotels, causing the loss of 500 beds in 2005 alone (municipal message, 26 July 2006), and a consequent decline in tourists that led to a decrease in related jobs of 4.5% between 1995 and 2005. The economic crisis highlighted the central and driving role of the festival for the city and contributed to bringing to the fore the need for infrastructural investments.

The combination of these two contingencies relaunched a debate about the opportunity to provide the festival with a proper ‘house’. Therefore, in July 2006, the mayor started the political process and public debate that led to the construction of, what today is known as, the PalaCinema.

### Data collection

Municipal documents and media articles published between 2004 and 2019 constitute our main source of data. We selected this temporal bracket to monitor the evolution of the PalaCinema project from the first claims highlighting the need for a festival house to the post-inauguration phase.

We retrieved 818 documents from the municipality website (www.locarno.ch), including municipality messages (MM), council minutes (CM) and commission reports (CR). These documents were authored by a set of actors representing powerful voices in the discursive city’s ‘identity moulding’ (Paasi, 2013: 1214). In particular, MMs and CMs represent the debate within the municipal government, including the voices of mayors with their majority and the minority parties. The same party was elected for the entire study period, with the first mayor resigning for personal reasons in 2015 and being replaced with a party colleague, who was subsequently officially elected in 2016. Meanwhile, CRs are technical-informative reports that also include voices of consultants, such as urban planners, architects, funders and associations. In addition, we retrieved 534 articles mentioning PalaCinema from the public archives of two local newspapers: *Corriere del Ticino* (CdT) and *La Regione* (LR). Media articles offer insights into the identified actors’ public posture ‘within the broader public arena’ (Taylor et al., 2014: 9) as well as the public postures of other powerful voices in moulding city identity discourse, such as local opinion leaders (e.g. journalists, citizen associations) and actors from the audio-visual world (e.g. LFF executives).

Finally, to complement the textual analysis with a more contextual understanding (Fairclough, 1992), we collected data through interviews with local opinion leaders (*n*=33), observations of public talks related to PalaCinema (*n*=7), and historical books (*n*=6). Table 1 summarises the data collected.

**Table 1.** Data collected and their use in the analysis.

Data source	Detail	Use in the analysis
Municipality documents	2004–2019 Municipality messages (MM) and Commission’s reports (CR) (713) Council minutes (CM) (105) Municipality messages are the texts prepared to announce and propose the themes under discussion during council meetings. Before the meetings, municipality commissions examine the messages and express their opinions through detailed written reports. After examining messages and reports, members of the municipality discuss and vote on decisions during the council. Under the label of municipal messages, the Locarno website also publishes strategic planning documents (such as the three-year action guidelines and financial plans).	Main object of analysis. Understanding evolution and interaction of meanings about Locarno identity and PalaCinema within the Municipality debate.
Media articles	2004–2019 *Corriere del Ticino* (289) and *La Regione* (245)	Main object of analysis. Understanding evolution and interaction of meanings about Locarno identity and PalaCinema through the public postures of Municipal governors and other powerful voices, such as journalists, associations and actors from the audio-visual industry.
Official public talks	2016–2018 Talks by politicians and representatives of the municipality, canton and confederation during events related to the festival and/or PalaCinema (7). Recorded.	Complementary object of analysis.
Semi-structured interviews (33)	Transcribed (one to two hours): 3 politicians, 1 member of regional development authority, 3 local businessmen, 2 PalaCinema technical consultants, 2 local journalists, 6 citizens and 16 festival employees plus several ethnographic conversations during observations	Complementary analysis. Understanding contextual interpretations of city identity. Understanding the urban context and multiple stakeholder positions.
Historical books on the city of Locarno and on the festival	6	Complementary analysis. Understanding history of Locarno
Observations	(About 50 hours) Municipality meetings, PalaCinema planning meetings (non-participant) PalaCinema opening during LFF 70 in 2017 (participant) PalaCinema official inauguration (participant)	Complementary analysis. Understanding the relationship among the municipality, the LFF and the evolution of urban projects related to the festival.

### Data analysis

We conducted a qualitative analysis of municipal documents and media texts. We coded our data through a multiple-step procedure (Gioia et al., 2013). In the first step of the analysis, the authors and one research assistant read through the data to identify those parts relevant for our purposes, regularly comparing emerging codes and fine-tuning the search for relevant elements. We coded all relevant text describing Locarno features, values and character, tracking whether these referred to the present (e.g. collective self-descriptions, current values, similarities/differences to other cities), the past (i.e. collective memories) or the future (i.e. aspirations). Similarly, we coded all references to PalaCinema (including earlier projects under different names) in terms of both the project evolution (e.g. decisions on location, financing, architectural properties) and meanings attached to it (e.g. a house for the festival, a place for audio-visual education, a commercial movie theatre). This initial coding enabled us to identify moments in which past, present and desired city identity meanings were mobilised more frequently in the texts, which coincided with turning points related to the PalaCinema project. Following up on this finding, we narrowed our focus to how PalaCinema developments and references to city identity appeared together; we represented these co-occurrence patterns under first-order categories labelled with words very close to our data.

In the second step, we aggregated those first-order categories into second-order themes describing co-occurrence patterns in discourse using more abstract general terms, describing the mechanisms through which past, present, or future identity meanings were mobilised in relationship to the PalaCinema project. For example, we aggregated first-order categories such as ‘iconic building project awakens Locarno (no longer inconclusive)’, ‘thanks to PalaCinema, Locarno is not “the poor sister” of the region’ and ‘PalaCinema shows that Locarno is the film festival’s home’ under the theme *foregrounding positive distinctive identity meanings.*

**Figure 1.**
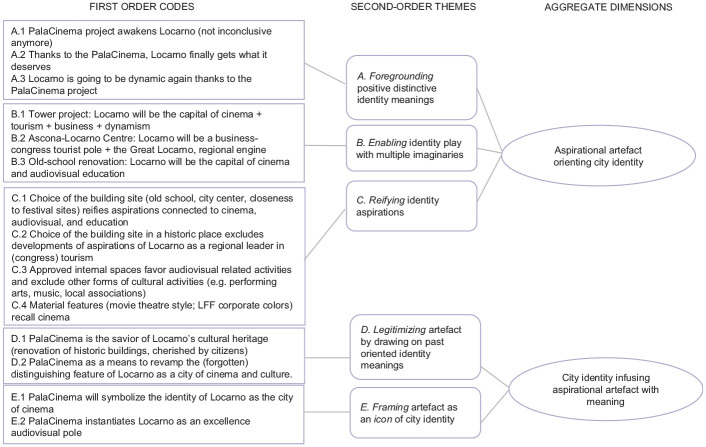
Data structure.

Third, we compared and contrasted the emerging themes with theories of discursive identity construction and aggregated them under more theoretical dimensions. Under the dimension *aspirational artefact orienting city identity*, we aggregated the three mechanisms describing how the PalaCinema, while in the making, performed an aspirational role in identity discourse. We borrowed the term ‘aspirational’ from performative discursive studies that define ‘aspirational identities’ (Thornborrow and Brown, 2009: 362), or ‘aspirational talk’, as discursive formulations expressing ‘productive idealizations’ that cannot yet be enacted but are brought into existence by declaring the intent to pursue them (Christensen et al., 2013: 379). By comparing and contrasting our data with those theories, we defined an aspirational artefact as an object that, during its materialisation, is mobilised in discourse to *orient* city identity idealisations. Similarly, by comparing and contrasting emerging mechanisms with the literature describing how planners infuse their material constructions with meanings (Comi and Whyte, 2018; Petani and Mengis, 2016), we aggregated the two remaining mechanisms under the dimension *city identity infusing aspirational artefact with meaning.* Figure 1 provides a visual representation of the data structure emerging from the analysis. Table 2 illustrates how the codes and themes are supported across different voices in the database.

**Table 2.** Themes and analytical codes: Overview of evidence across different voices.

	Mechanisms	Majority party voices	Minority parties’ voices	Opinion leaders (architects, journalists, audiovisual actors)
Aspirational artefact orienting city identity	A. *Foregrounding* positive distinctive identity meanings A.1 PalaCinema project awakens Locarno (not inconclusive anymore) A.2 Thanks to the PalaCinema, Locarno finally gets what it deserves A.3 Locarno is going to be dynamic again thanks to the PalaCinema project	A.1 Strong evidence in Municipal documents, and complementary interviews A.2 Strong evidence in Municipal documents, the media and complementary interviews A. 3 Moderate evidence in Municipal documents and complementary interviews	A.1 Moderate evidence in Municipal documents and the media A.3 Moderate evidence in Municipal documents	A.1 Strong evidence in the media A.2 Strong evidence in the media A.3 Moderate evidence in the media
	B. *Enabling* identity play with multiple imaginaries B.1 Tower project: Locarno will be the capital of cinema + tourism + business + dynamism B.2 Ascona-Locarno Centre: Locarno will be a business-congress tourist pole + the Great Locarno, regional engine B.3 Old-school renovation: Locarno will be the capital of cinema and audio-visual education	B.1 Moderate evidence in the municipal documents B.2 Moderate evidence in the municipal documents and the media B.3 Strong evidence in the municipal documents and the media	B.1 Weak evidence in municipal documents	B.1 Strong evidence in the media B.2 Moderate evidence in the media B.3 Strong evidence in the media
	C. *Reifying* identity aspirations C.1 Choice of the building site (old school, city centre, closeness to festival sites) reifies aspirations connected to cinema, audio-visual and education C.2 Choice of the building site in a historic place excludes developments of aspirations of Locarno as a regional leader in (congress) tourism (absence of aspirations about business tourism after 2010). C.3 Approved internal spaces favour audio-visual-related activities and exclude other forms of cultural activities (e.g. performing arts, music, local associations) C.4 Material features (movie theatre style; LFF corporate colours) recall cinema.	C.1 Strong evidence in municipal documents and the media C.2 Negative evidence (those claims fade out) C.3 Strong evidence in municipal documents (including approved plans) C.4 Moderate evidence in municipal documents (including approved plans)		C.1 Strong evidence in the media C.2 Negative evidence (those claims fade out) C.3 Moderate evidence in the media and municipal documents C.4 Moderate evidence in the media and municipal documents
City identity infusing aspirational artefact with meaning	D. *Legitimising* artefact by drawing on past oriented identity meanings D.1 PalaCinema is the saviour of Locarno’s cultural heritage (renovation of historic buildings, cherished by citizens) D.2 PalaCinema as a means to revamp the (forgotten) distinguishing feature of Locarno as a city of cinema and culture.	D.1 Moderate evidence in municipal documents and the media D.2 Strong evidence in municipal documents and the media, and complementary interviews		D.1 Weak evidence in the media and complementary interviews D.2 Strong evidence in the media
	E. *Framing* artefact as an icon of city identity E.1 PalaCinema will symbolise the identity of Locarno as the city of cinema E.2 PalaCinema instantiates Locarno as an audio-visual pole	E.1 Strong evidence in municipal documents and the media E.2 Strong evidence in municipal documents and the media	Against E.1 and E.2 Moderate evidence in municipal documents and the media	E.1 Strong evidence in the media E.2 Strong evidence in the media

For the sake of clarity, we describe the analytical steps in a linear manner; however, passages from one step to the other were by no means clearly demarcated, and the whole analysis required multiple iterative loops across the steps. Furthermore, in all three steps we complemented the findings obtained through textual analysis with contextual insights gleaned from observations, interviews and historical books. All coding was conducted using the software NVivo11.

## Findings

Before providing a narrative account of the two dimensions and underlying themes emerging from the analysis, we briefly illustrate the timeline of the PalaCinema planning and its turning points.

### Project timeline

As outlined in the case description, a debate about the need to provide a permanent house for the festival emerged strongly in the early 2000s, although the subsequent conversation did not unfold in a linear and steady way. Rather, it was characterised by periods of heated debates and relatively silent ones.

A first turning point emerged when the municipality relaunched the ‘undelayable construction of a permanent structure’ to support the important film festival and definitively anchor it to Locarno, leading private and public actors to propose multiple projects in 2006 and 2007. Two were discussed and pre-approved by the local government: a Tower of Cinema and a Congressional Palace in a joint venture with the neighbouring town of Ascona. A second turning point happened in 2009, when the municipality decided to locate the festival house in an existing historical building in the heart of the city and renovate it (old school building; final approval in MM, 19 January 2010). A third turning point stretches from the actual kick-off of the project thanks to a donation (10 million Swiss francs) from a private foundation (a non-profit organisation funding socio-cultural projects) in 2012 and the opening of the construction site in 2014. The donor required respect for a tight construction timeline to favour the ‘quick realization of an all-too-expected facility’ (CM, 16 July 2012), and that the building should contain three movie theatres as requested by LFF. This precipitated the public tender procedure and the approval of the architectural renovation project, but also raised opposition, with minority parties calling for a referendum, although it did not achieve enough citizens’ signatures to be launched, thereby paving the way to proceed with the work. The building was finally inaugurated in 2017.

### Aspirational artefact orienting city identity

The three mechanisms we describe below show how the PalaCinema project, at different stages of development, performed an aspirational role by (re)orienting various actors’ city identity articulations.

#### Foregrounding positive distinctive identity meanings

Throughout the period under analysis, and most frequently during the first turning point (2006–2007), the project to build a house for the LFF was mobilised as a concrete means to foreground positive distinctive identity meanings and thus get rid of those negative meanings both citizens and external actors associated with the city’s identity, including being a decayed city with an inconclusive and contemptuous character (Corriere del Ticino, 2006) that makes it impossible to be constructive and often leads to the use of epithets such as the ‘poor sister’ or the ‘hole of the donut’ when compared to neighbouring cities (majority party politician, 2017, personal communication). We identify this foregrounding mechanism as a commonality in various actors’ voices, even within the minority parties in the city’s government. We saw it playing out in two ways. First, the house project could articulate Locarno’s identity by emphasising the distinctive and prestigious trait of being the location of LFF, thereby leveraging collective pride, prestige and internationality. Municipal documents often report that building a house for LFF would indeed realise ‘what Locarno deserves’ (CM, 17 September 2007) and expand the spirit of ‘incomparable cultural vitality’ and ‘openness and internationality’ (MM, 26 July 2006), not only during LFF, but all year long. Accordingly, journalists and local opinion leaders welcomed the decision to engage in the project as a solution to the ‘embarrassing *impasse*’ of being ‘Locarno city of cinema, Locarno without movie theatres’ (IC, 2006b).

Second, the project was often referred to as a ‘magic wand’ (local politician, 2017, personal communication), a game changer that ‘is awakening’ Locarno after many failed promises (about the festival’s house and other relevant projects) that never achieved serious commitment from the city municipality. This awakening was achieved by both the initial project proponents (e.g. the majority party, in praise of their work) and other voices typically more critical of cultural projects, such as representatives of a minority right-wing populist party, with claims such as ‘Cinderella [Locarno] is getting back on her feet thanks to projects like the PalaCinema and eventually decided to go to the court ball’ released to the local media (*La Regione*, 2006). In other words, the project enabled the government to signal that ‘the classic affirmation that Locarno always misses the boat needs to be solemnly denied’ (CM, 18 June 2007).

#### Enabling identity play with multiple imaginaries

Before the approval of the final PalaCinema project, multiple project ideas were discussed by the city government and debated in the media. Data show that the different features of each specific project (i.e. location, type of building and intended use) allowed actors to play with multiple city identity imaginaries, qualifying in very different ways the aspiration to become ‘the capital of cinema’, thanks to the construction of the PalaCinema (IC, 2006a).

For instance, on the occasion of the first project proposal of a tower, changes to the town map were approved (MM, 26 July 2006) and engineers consulted to estimate the cost for the construction of a 70-m tall building near the city centre. The project included movie theatres, a luxury restaurant and a hotel. When illustrating and discussing this project, municipal representatives, journalists and opinion leaders associated the expectations for a prestigious, impressive building with identity aspirations, qualifying Locarno as international and dynamic, both in the media (Gianetti Lorenzetti, 2006) and in council meetings, as explicitly reported in the following quote:

the PalaCinema will be the tallest building in the canton – a feature that is sufficient to burden the developer with the responsibility of building a fine structure that will become an international symbol of the dynamism of contemporary Locarno, the capital of Swiss cinema. (CM, 18 September 2006)

The tower project was dismissed when the mayors of Locarno and Ascona signed an agreement in 2007 declaring the intent to engage jointly in the construction of a Cultural Congress Cinematographic Centre in the common dismantled area of a former airport. The project idea, inscribed in the broader debate for an urban aggregation project at the regional level (Corriere del Ticino, 2007), eventually failed, but it enabled representatives of the majority party to formulate rhetorical dreams of a ‘Great Locarno’ as a regional engine and cantonal congressional tourist destination (CM, 17 December 2007). The traditional cantonal identity feature of Switzerland’s *Sonnenstube* (sunny living room) was also frequently recalled when talking about Locarno in association with Ascona, which never lost its appeal for Swiss-German tourists (CM, 7 May 2007). Imaginaries and aspirations related to grandeur and congressional tourism rapidly disappeared together with the failure of the Tower and the Locarno–Ascona projects.

With the identification of a physical space – part of the Locarno cultural heritage cherished by many citizens, according to media articles and several interviewees – a clear rhetorical shift emerged in municipal documents re-claiming Locarno as a city of culture (CM, 27 April 2009), foregrounding the aspiration to become an audio-visual educational pole.

#### Reifying identity aspirations

The winning architectural project in 2012 gave a material form to PalaCinema. Chosen from among 80 projects, the winning one was quite a conservative renovation project. It showcased cinema due to its movie theatre structure (foyer-style entrance, three movie theatres), but also referenced LFF thanks to the colours that were reminiscent of its brand. No features recalling the other cultural activities or spaces of the former inhabitants of the building (a local choir, various artistic associations) were included (MM, 10 July 2013). Instead, spaces for cinema archives, audio-visual schools, media and public entities attracting film shoots in Locarno were included in the project, together with the LFF’s permanent headquarters. These planning decisions, designed primarily for audio-visual performance, production and education, silenced previously discussed alternatives in terms of not only the material form of the building, but also related identity aspirations, as shown in this declaration to the media by the LFF president, who is also a member of the PalaCinema foundation:

Among projects submitted there were some that would revolutionize the city, but there was no explicit desire to make Locarno a Bilbao. The chosen project enhances the building concerned without changing it more than needed and allows the two fundamental aspects that characterize it to be integrated in an entirely optimal way: cinema and school. (Conti, 2013: 17)

Therefore the choice of a material location, part of the city’s historical heritage laden with an affective legacy, silenced aspirations to hypermodernity (as foregrounded by the former tower project), while the approved architectural project reified the aspiration that ‘the infrastructure will finally make Locarno able to become a national and international formative pole in the audio-visual field’ (Pelloni, 2013: 17).

### City identity infusing aspirational artefact with meaning

Thus far we have shown how the evolving material features of the project (plans, locations, design, allocated spaces) influenced the discursive articulations of identity meanings in the public debate. We now describe how actors infused the evolving project with identity meanings taken from collective memories, present understandings and evolving future aspirations.

#### Legitimising the artefact by drawing on past oriented identity meanings

Our data show how features referring to the past were used to infuse the project with meaning, especially when needing to legitimise it in the public debate. As shown in the previous section, the need to invest in an infrastructure was initially discussed considering the need to support the LFF and to overcome the lack of screening venues. Therefore, in the public debate, the lack of adequate infrastructures for the film festival was equated to betraying a distinctive central historical feature of the city identity, such as being ‘the quintessential City of the Film Festival’ (IC, 2006c: 23).

However, the rapidly changing projects, while enabling multiple imaginaries as previously illustrated, also raised a few sceptical voices delegitimising the ambitious magnificence of some plans which were clashing with the current identity feature of inconclusiveness as perceived by locals. While expressing pride and approving the need to anchor LFF to the city, citizens voiced concerns through the media about the ‘usual delusions of grandeur’ and politically ambitious (but not realisable) promises (IC, 2006b), talking also of ‘yet another Locarno utopia’ (Bacchetta Cattori, 2006). For example, in a letter commenting on the tower project, a local architect ironically questioned whether a shorter Eiffel Tower – actually, a quarter of it – ‘could really be the longed-for emblem’ of ‘the film festival (and Locarno tout court)’ (Ulmi, 2006).

The decision to renovate the old school building was eventually legitimised by the city government, with arguments related to the importance of culture in Locarno’s past and framing the approved PalaCinema as the saviour of Locarno’s cultural heritage by providing ‘value in terms of identity for Locarno’s inhabitants’ (CM, 27 April 2009) within a more general frame related to preserving the city’s historical identity, as illustrated in the following excerpt:

The municipality foresees giving priority to some projects of regional interest that will give important input to the development of the Locarnese. In line with this, one of the main projects is the PalaCinema, to which the valorization of the monumental area is strictly related. (Financial plan 2009–2012, 19 January 2010)

Project supporters mobilised not only LFF as a distinctive feature of the past Locarno identity, but also the general collective (and almost lost) memory of Locarno as a city of culture. Municipal documents drafted by the majority party often refer to a past in which Locarno was a city of international cultural debate and innovation. Having been a city of culture in the past justifies the need to invest in the PalaCinema project for the future, as shown by this excerpt from the majority committee report:

Culture counts, culture has weight, culture matters […] Locarno has all the characteristics to aspire to become a cultural centre (with a focus on visual arts); this claim is not only supported by the long-standing tradition of the festival, but also by all the other activities that marked the history of this place. […] We have to thank several artists, art patrons, and visionary people for this. The PalaCinema is a rare opportunity to add our name to this list also. (CR, 11 March 2013)

Culture is often used together with a welcoming feature characterising Locarno (as a city of tourism and the peaceful home of international treaties) to distinguish it from other cantonal cities and to infuse project-related investments with identity-relevant meanings, as this quote by a Locarno mayor exemplifies:

Locarno is not a financial centre like Lugano or an administrative city like Bellinzona; we are a city that has high-quality and excellent tourism and that provides the canton with multifaceted culture. We therefore need to invest in culture and in the infrastructures necessary to make it thrive. (CM, 16 July 2012)

The final approved project was legitimised by proponents as something ‘to restore the strength and splendour of an architectural object such as the former school building, with which Locarno’s collective memory and feelings are interwoven’ (Broggini, 2012), thus revamping and justifying Locarno’s collective memory, resolving accusations of grandeur and utopia voiced by opinion leaders and citizens in the media in response to previously proposed projects.

#### Framing artefact as an icon of city identity

What was initially simply a project to provide infrastructures to LFF soon transformed into the planning of an iconic building, not just for its visibility and design, but also because actors started framing it as an icon representing the identity of the city (i.e. a part representing the whole in a synecdoche relationship). Initially, even before knowing the location, shape, size, or financial value of the house for the festival, the project was infused with positive identity meanings related to being home to the LFF – ‘the facility that would make Locarno the capital of cinema’ (Pettinati, 2006). Local politicians and journalists debated the situation with a sense of anticipation: ‘Will PalaCinema become the new symbol of Locarno, replacing the Madonna del Sasso [historical church], traditionally associated to the city image? Somebody already believes this will happen’ (IC et al., 2006).

With the emergence of imaginaries linked to multiple proposals, project proponents framed PalaCinema as a Locarno icon, infusing it with different connotations related to being the city of cinema: the tower being ‘the door of the city’ and ‘the symbol of the new Locarno’ (CM, 18 September 2006), the Congress Centre an icon of the ‘Great Locarno’ and a ‘regional engine’ (CM, 17 December 2007), and later the approved PalaCinema an icon of Locarno as an ‘audio-visual and cinematographic pole’ (Rizzi, 2014). Claims that PalaCinema would represent Locarno as a place of ‘innovative and high-quality specialization’ (MM, 22 June 2012) intensified in the public debate and infused the decisions about the evolving material shape of the building with identity meaning. For instance, architectural plans were often taken up by politicians and inscribed with additional meanings detailing how PalaCinema spaces could be potentially allocated in order to make Locarno an audio-visual pole. For instance, spaces for film archives, equipped rooms for audio-visual professional education, and a newly founded film commission were approved in the plan and associated with the desired city identity.

However, this iconisation was also resisted by a few critical voices. The controversy peaked just after receipt of the private donation kicking off the project in 2012, characterised by opponents as a ‘poisoned gift’ (local politician, 2017, personal communication), that triggered an intense debate within the local government and the media as well as a referendum proposal calling for opposition to the project by two minority parties. Ultimately, not enough signatures were gathered to launch the referendum; however, the referendum campaign in 2013 was a moment in which we could observe various voices (politicians, journalists, intellectuals, citizens’ associations) explicitly discussing in the media whether a PalaCinema with those emerging material features could represent the present and desired identity of Locarno. Opponents emphasised that a palace with screening theatres fully equipped to work with commercial film programming, could not be the icon of the city and ‘the only dispenser of culture’ (Tagliaferri, 2013), and would instead kill smaller movie theatres and other cultural features relevant to Locarno inhabitants (La Regione, 2013; Forni, 2013). On the contrary, supporters convincingly affirmed that movie theatres were essential for the festival; they said that PalaCinema was much more than that and framed it as ‘a catalyzer’ and an ‘engine’ allowing for ‘multiple synergies’ (Conti, 2013) that would clearly distinguish the Locarno profile around cinema and video-making (La Regione, 2014).

However, with the failure of the referendum and the actual beginning of construction, aspired identity claims about Locarno becoming an audio-visual pole gained traction in the public debate and became more consensual across multiple voices until inauguration.

Although the contextual knowledge we derived from complementary data (interviews and participation to events) suggests that many local actors (businesspeople, journalists, common citizens) were not persuaded that PalaCinema would change Locarno in the short term, a sense of pride prevailed at inauguration (authors’ observation notes), where local administrators equated the realisation of the building with the realisation of the desired identity, welcoming PalaCinema ‘as an expression of our creativity and professionalism’ (Mayor, Lo Russo, 2017).

## Discussion

Our findings provide a novel understanding of the anticipation effects of iconic buildings in the making by showing their role as aspirational artefacts in city identity discourse. In particular, the PalaCinema case shows that this role comprises reciprocal interactions of mechanisms through which the building orients city identity discursive articulations by urban actors as well as mechanisms through which actors infuse the evolving materiality of the building with identity meanings. First, these reciprocal interactions allowed for reconciling a glorious past with a present rather blurred city identity. The envisioned project, in fact, foregrounded positive city identity meanings, thereby boosting pride, while concomitantly past-oriented identity meanings drawn from collective memory allowed for discursively legitimising the project itself.

Second, the iconic building project activated city identity future-making by opening up the possibility of playing with multiple imaginaries and then closing down the range of possible choices by reifying only some desired meanings in its progressively materialising features. This process was reinforced by urban actors continually framing the evolving project as an icon not only of present city identity perceptions, but also of identity aspirations.

We next discuss these mechanisms and their reciprocal interplay. In particular, we discuss the role of aspirational identity artefacts as temporal bridges in city identity discourse and how this role plays out in the relationship between city identity and urban material transformations.

### Aspirational artefacts as temporal bridges

It is by now almost taken for granted that iconic buildings are important symbols of a city’s identity (Jones and Svejenova, 2018; Kavaratzis and Hatch, 2013) as well as inspirations for meaning negotiations and development of futuristic imaginaries with their visual and material stance (Gospodini, 2004; Jencks, 2006). They have been defined as ‘enigmatic signifiers’ (Jencks, 2006: 4) as their often unusual shapes and famous architects' hallmarks offer themselves to multiple interpretations, as suggested by dominant urban voices or users’ perceptions, and are often objects of debate and contestation (Gospodini, 2004; Jencks, 2006; Sadana 2018).

Our case extends this debate in two ways: first by discussing the futuremaking semantic potential of iconic buildings while in the making and second by problematising the notion of iconic and proposing that major architectural projects may be not only icons of a generic hypermodern urbanity, but also buildings functioning as temporal bridges between very distinctive collective memories and future aspirations.

As a first point, our case shows that iconic buildings in-the-making are enigmatic signifiers of what a city – through various actors’ voices – aspires to become. We noticed that, at the very least, planning an iconic project activates a collective reflection about a city’s identity core and distinguishing features, with decision makers and planners mobilising city identity features every time the project reaches a turning point. However, this was not circumscribed to decision-making rooms in the municipality, but always expanded to debates in the media. By reporting politicians’ and planners’ voices, journalists and other opinion leaders often commented on the project and mobilised their own interpretation of the city identity, at times leading to public debate and deliberation about ‘who we are’ and ‘who we are not’.

By offering an enigmatic signifier – albeit in its pre-material potency state – the iconic building project enables actors to mould identity aspirations, not just as abstract desired identity claims, but as ‘productive idealizations’ (Christensen et al., 2013: 379) brought into existence by the progressive material development of the project. Such aspirations provide short-term intermediary confirmations that a renewed ‘imagined community’ (Anderson, 1991) is viable in the long run. Therefore, although iconic buildings under construction have some ‘anticipation effects’, such as attracting economic resources and reconfiguring demographics and social status expectations in their area (Raevskikh, 2018: 93; Sadana, 2018), our findings show that their anticipatory role also consists of aspirational power in (re)orienting urban actors’ ability to imagine and negotiate a shared desired city identity. In this regard, our findings confirm and extend the notion that iconic buildings are future-oriented monuments (Jencks, 2006; Krzyżanowska, 2016), not only for the future-oriented semantic power of their material posture in the urban landscape, but also because the very act of building them and negotiating their function forces future-making activities.

As a second point, our case allows us to appreciate that iconic buildings may not only be monuments to the future, but also a bridge connecting collective memories and future aspirations. Discussions around the PalaCinema project helped recover features of the Locarno past that were no longer as salient, at least in guiding public policymaking, as representatives of the city government themselves admitted. In our case, the iconic building helped uncover a glorious past that had become blurred and transform it in future aspirations.

Thus, iconic projects may serve as temporal bridges insofar as they force the engagement to reflect on the threefold temporal orientation of city identity discourse: urban actors can infuse the developing project with meanings of the past that preserve the semantic representation of the city’s unique features, even in a future-oriented aspirational declination. In this way, iconic buildings are no longer just highly visible symbols of a particular futuristic architectural style.

We use the term bridge based on the mechanisms emerging from our study; however, we can speculate that the bridging may instead manifest in an voluntary detachment from the past, as the cases of little Shanghai in traditional Chinese cities (Anzoise et al., 2020) and Delhi’s metro project (Sadana, 2018) seem to suggest. Even if not bridging, in these cases engagement in aspirational projects also enabled a temporal reflection on city identity.

In sum, although literature acknowledges that at various levels collective identity discourse has a threefold temporal dimension – namely, being concomitantly past, present and future oriented (Anderson, 1991; De Cillia et al., 1999; Schultz and Hernes, 2013; Thornborrow and Brown, 2009) – our study provides a further understanding of how engaging in the planning of an important project can support the (re)connection of these temporal orientations in present identity articulations. At the same time, it shows that different temporal identity orientations play particular roles in enabling or harnessing certain advancements of the project, as we discuss next.

### Role of identity in urban material transformations

As a second area of contribution, our findings advance our understanding of the relationship between city identity and urban material transformations. Existing literature often tangentially discusses that identity matters in urban change, using differentiated definitions of what city identity exactly means, but often understanding it under an institutional theory lens foregrounding the role of values and broader societal discourses (Fahrat, 2015; Leixnering and Höllerer, 2022). Here we propose that the way actors discursively claim and reclaim a city identity’s unique and distinctive features (not only values, but also characteristics, memories and aspirations) has a harnessing role on the aspirational power exercised by projects for relevant material urban transformations (i.e. not only in its limiting sense, but also in the sense of guiding it). Imaginaries activated by building plans, which were too detached from past and present city identity features, were criticised in the public debate and ultimately did not take off. In our case, this identity-harnessing effect was particularly visible when pre-material instantiations of project proposals opened up, captivating imaginaries in line with mega trends in urban plans (hypermodern architecture; creativity; luxury). Although proud about the decision to actually engage in iconic building planning, various actors also mobilised current and past identity meanings to criticise those projects as unsuitable for the city. On the contrary, the fact that the old school building renovation was infused by planners with meanings linking the project to the past, and not only to a desired future, allowed them to actually recompose a consensual voice across opinion leaders and associations, supporting the aspiration to become an audio-visual pole moulded by the local government, despite minority parties’ strong opposition. In other words, current city identity understandings may allow the development of aspirations that can resonate with a city’s history, protecting projects from the risk that suggest overly divergent interpretations of a city (Anzoise et al., 2020).

This confirms and extends what Leixnering and Höllerer (2022) proposed on the relationship between city identity and its structure. Although one building, even if iconic, does not change the socio-material structure of a city per se, it undoubtedly sparks transformational dynamics. Our case showed how the reciprocal interplay between the evolving material features of the project and identity mobilised in discourse to infuse them with meanings enabled Locarno to engage in adaptive resilience rather than transformational change. This was not just a matter of socio-political values, as discussed by Leixnering and Höllerer (2022), but also of maintaining collective distinctive features while pursuing broader normative urban development ideals of creativity and innovation.

This is a particularly relevant insight from our case because the project was not inscribed in a broader strategic exercise of city envisioning and branding, which have been proved to activate collective reflections and the craft of strategic narratives to define (and sell) the unique character of a city, as shown by the Sydney 2030 case, for example (Kornberger et al., 2021). Rather, the initial very functional decision to support the local festival with proper infrastructure transformed, over time, into something identity defining, leading to envisioning exercises long after the end of our empirical observation with the launch of the Locarno Media City project in 2019. This leads us to agree with scholars asserting that planning and strategising (Kornberger et al., 2021; Paasi, 2013) favour the emergence of a manifest sense of community and identity. We add that single projects, which are demanding for urban planning but not necessarily launched as pieces of a larger strategic effort, may become relevant battlefields (Paasi, 2013) for city identity negotiations. Previous identity understandings, although dormant for a number of reasons, can harness the directions of plans and strategising. Indeed, our case shows how engaging in the planning of a single important project forced people to remember that Locarno was a community with certain distinctive features and collective pride, suggesting that past and current city identity meanings contribute to avoiding flights of fancy in the moulding of identity aspirations. At the same time, they contribute to legitimising and supporting project features that allow communities to stay true to their core and distinguishing identity features, while heading towards new directions that still have a relationship of continuity instead of breaking with the city’s past.

As is inherent to single qualitative case studies, our findings need replication. Locarno’s particular situation as a very small and provincial city hosting a large, internationally renowned event may have over-emphasised the role of the aspirational identity artefact. In addition, the opacity and ambiguities in the Locarno identity discourse, combined with the success of the festival in a context of negative economic conjuncture when the project started to be discussed, surely provided fertile terrain for the aspirational potential of the envisioned object. In particular, this initial context may have overemphasised the role of the project in foregrounding positive identity meanings (at the expense of negative ones) and boosting pride. Despite this, we believe the mechanisms we identified provide a useful bedrock for comparison with other cases. It would be interesting, for instance, to investigate the aspirational role of iconic buildings in-the-making in the context of larger cosmopolitan cities and perhaps compare how multiple parallel projects interact in a city’s identity discourse, by reconciling or disjoining cities’ and neighbourhoods’ identities.

Moreover, the relevant role that the developing material features of the project proved to have in identity discourse suggests the need to consider more closely in future studies the appropriate material aspects of aspirational identity artefacts, such as through ethnographic socio-material approaches (Amin and Cirolia, 2018; Petani and Mengis, 2016).

Finally, research has usually considered urban buildings as a manifestation of city identity (Jones and Svejenova, 2018) or a suggestion for futuristic interpretations detached from the current identity of the city (Gospodini, 2004; Jencks, 2006). Instead, our case shows that PalaCinema eventually ended up being a manifestation of not-yet-realised aspirations. In this sense, future research may further explore the aspirational role iconic buildings maintain after their construction, whether they succeed in actually transforming aspirations into lived city identity features or keep alive a dream that never comes true.

## Conclusion

Investments in iconic buildings have increased in recent years, together with debates and mixed views about their actual urban regenerating power. In many cases, they proved to increase economic and creative/knowledge workers’ inflow – the renowned Bilbao effect (Jencks, 2006; Raevskikh, 2018); however, many others raised concerns linked to, among others, the loss of a city’s unique identity and over-adaptation to normative globalised pressures for creativity, liveability and modernity (Anzoise et al., 2020; Nagel and Satoh, 2019; Sadana, 2018). Our study contributes to this debate by proposing that iconic buildings may serve as aspirational identity artefacts while in-the-making and by unpacking the mechanisms of their anticipation role, which show that the planning of an iconic building can be a productive ground to reflect, re-orient and re-claim the unique features of a city’s identity while aspiring for a different future.

In practice, our study seems to suggest a counterpoint to the general view of speculations in urban planning and to the often criticised empty, useless architectural projects in response to modern urbanisation global pressures (Nagel and Satoh, 2019; Scott, 2014). Indeed, this is not a normative or unproblematic consideration, and we acknowledge that this is not always the case; gaps may emerge in conflictual interpretations and (dis)identification to aspirational artefacts by different stakeholder groups, which merit further investigations. However, we believe that, in an era in which urban physical transformations are pervasive and global forces are pushing towards mainstream interpretations of urban space design, it is important to understand how material transformations interact with collective self-definitions. We believe future studies in this direction may help in the broader endeavour of understanding how cities preserve their uniqueness while pursuing global urban development ideals.
